# HyperHealth: a pilot study on AI-driven COVID-19 detection using hyperspectral fingertip images

**DOI:** 10.1038/s41598-026-47056-x

**Published:** 2026-04-30

**Authors:** Emanuela Marasco, Shruti Wagle, Mason Rule, Katherine Lee-Wisdom, Maheen H. Khan, John Deeken, Raghavendra Ramachandra, Fatima Karzai, M. Pia Morelli, Charalampos S. Floudas, James L. Gulley

**Affiliations:** 1 Computer Science, Virginia Commonwealth University, Richmond, VA 23284 USA; 2 Computer Engineering, New York University, New York, NY 10012 USA; 3 Center for Immuno-Oncology, Center for Cancer Research, National Cancer Institute, National Institutes of Health, Bethesda, MD 20892 USA; 4 Office of Research Nursing, Center for Cancer Research, National Cancer Institute, National Institutes of Health, Bethesda, MD USA; 5 INOVA, Schar Cancer Institute - White Oak Clinic, Fairfax, VA 22031 USA; 6 Department of Information Security and Communication Technology, Norwegian University of Science and Technology, Gjøvik, 2815 Norway; 7 Department of Gastrointestinal Medical Oncology, The University of Texas, MD Anderson Cancer Center, Houston, TX 77030 USA

**Keywords:** Hyperspectral physiological images, Health monitoring, COVID-19, Biomarkers, Computational biology and bioinformatics, Diseases, Health care, Mathematics and computing

## Abstract

Despite the reduced impact of COVID-19 due to widespread vaccination and improved treatments, a critical need remains for accessible, scalable, and rapid screening tools to address current and future infectious disease threats. Hyperspectral Imaging (HSI) may be such a tool, but the current limited availability of data from COVID-19 positive individuals hinders traditional supervised learning approaches. To overcome this, we designed a novel framework that integrates HSI with Artificial Intelligence (AI) analysis for detecting COVID-19 status from images of fingertips, thereby demonstrating infection detection through biometric data. By analyzing high-dimensional spectral signatures from the fingertip, the approach identifies distinctive patterns linked to physiological changes caused by the virus. In this pilot study, a Support Vector Machine (SVM) and a Logistic Regression classification algorithm demonstrated high accuracy in classifying HSI images, underscoring the potential of hyperspectral features for non-invasive, real-time health monitoring, even with the limitations of a small dataset. We introduced the first publicly available dataset of HSI images from COVID-19-positive individuals. This contribution sets a foundation for advancing biometric spectral imaging in biomedical research and AI-powered diagnostics. The datasets used during this study is available from the corresponding author upon reasonable request.

## Introduction

The Coronavirus Disease 2019 (COVID-19) pandemic has posed unprecedented challenges to healthcare systems worldwide. Mass testing, contact tracing, and social distancing were strategies used to limit the spread of the virus at the beginning of the pandemic, prior to the development and widespread availability of vaccines. Mass testing for COVID-19 in the public health setting required rapid and accurate point-of-care tests that could be deployed at scale. Nasopharyngeal swab tests for viral gene detection by RT-PCR are the gold standard for COVID-19 testing, but they require sample collection by a healthcare provider, are uncomfortable and invasive for patients^1^, and results may take several hours. Rapid antigen detection tests require nasal swabbing, which can be self-administered, and produce qualitative results in 15–30 minutes^2^, an interval still limiting their applicability for mass testing. Therefore, a modality for point-of-care rapid, non-invasive, and scalable testing to facilitate screening at the population level remains a critical unmet need. COVID-19 triggers systemic inflammation, marked by elevated biomarkers such as Interleukin-6 (IL-6), C-Reactive Protein (CRP), Lactate Dehydrogenase (LDH), and D-dimer^3^. These metabolites can be secreted through eccrine sweat glands and detected on the skin surface, making Hyperspectral Imaging (HSI) a promising non-invasive diagnostic tool. HSI captures detailed spatial and spectral data across visible to near-infrared wavelengths (400–2500 nm)^4^. Unlike RGB imaging, HSI produces a ”hypercube” with two spatial and one spectral dimension, where each pixel contains a full spectral signature, enabling detection of subtle biochemical and physical tissue changes^5^. With this work, we seek to answer the question: *Can a HSI fingerprint region reveal COVID-19 infection?*. To test this hypothesis, we collected and analyzed hyperspectral fingerprint images from 53 subjects who had been previously tested for COVID-19 using the Standard of Care (SOC) assay.

The paper is organized as follows: We begin by presenting the experimental results, along with the scientific basis for detecting COVID-19-related features from hyperspectral fingertip data. We then describe the proposed methods and conclude with a discussion.

## Related work

Recent work has explored deep learning approaches to detect COVID-19 infection from medical imaging, particularly chest radiographs and CT scans, often using transfer learning to address limited labeled data and achieve high classification accuracy^6,7^. Although these modalities provide valuable diagnostic information, they can be limited by radiation exposure, lower sensitivity to early-stage physiological changes, and reliance on structural rather than biochemical signatures. In contrast, HSI offers a non-invasive and user-friendly alternative that captures rich spectral information beyond the visible spectrum, enabling the detection of subtle physiological and biochemical variations associated with disease. HSI is contactless, does not require ionizing radiation, and can provide more detailed and discriminative features, making it well-suited for early detection and continuous monitoring. Its ability to extract fine-grained spectral signatures enhances robustness in classification tasks, offering a promising complementary or alternative approach to traditional imaging modalities. The proposed work was motivated by the need for a point-of-care rapid, non-invasive, and scalable testing to facilitate screening at the population level. X-rays and CT imaging are tools with great utility for the diagnosis of pulmonary involvement in patients with symptoms of infection from COVID-19 or other pathogens, not appropriate for screening at the population.

## Results

This manuscript reports the results of the first application of HSI to detect COVID-19-related features through fingertip analysis. Furthermore, our findings highlight the potential role for integrating AI into clinical workflows to improve diagnostic testing^8^.

### Study design and patient characteristics

**Fig. 1:**
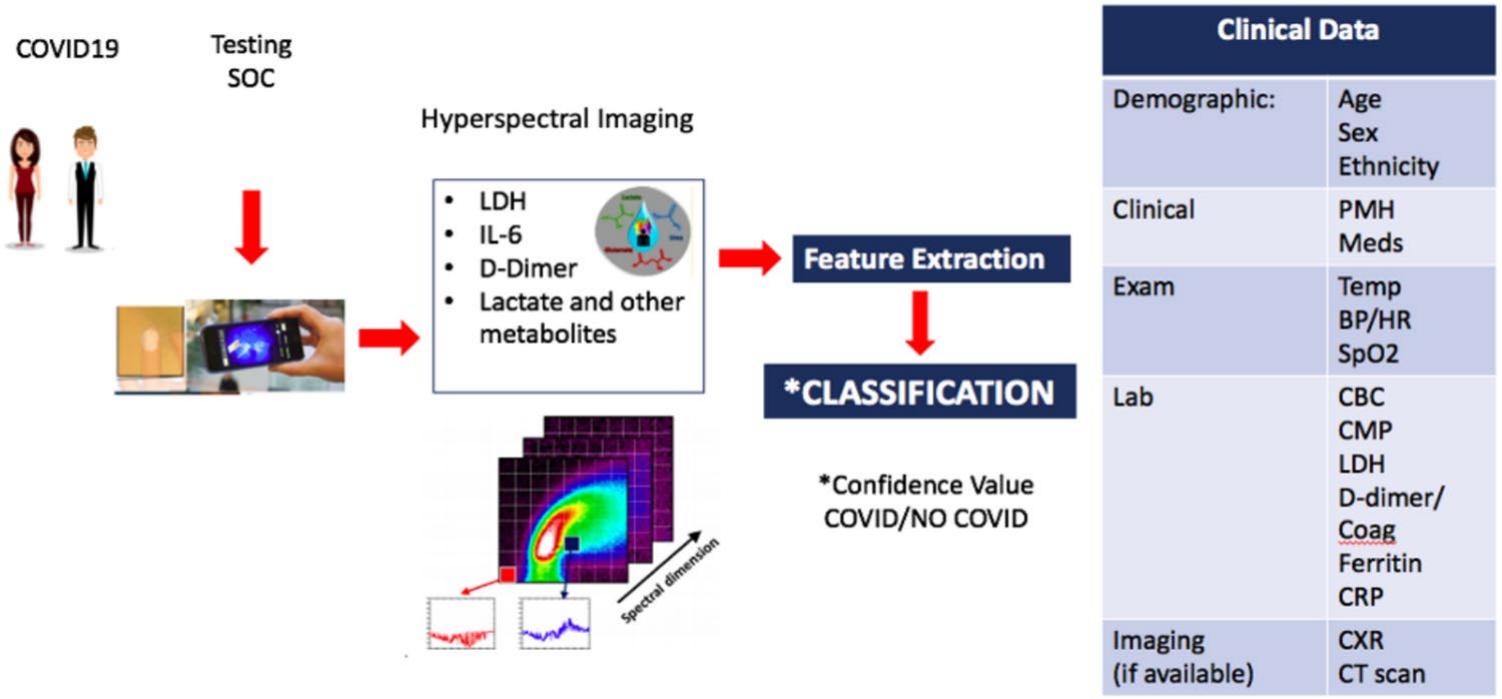
Information flow of the proposed COVID-19 detection system. Individuals present their fingerprints to allow the hyperspectral camera to capture detailed spectral data. The collected spectral signatures are processed and used to train a pattern classifier, which then outputs a real-time decision indicating the presence or absence of COVID-19 infection. As part of the overall study design, optional clinical data, including inflammatory cytokine panels and routine laboratory evaluations such as Complete Blood Count (CBC), chemistry, hepatic, and mineral panels collected within 7 days of enrollment, were also acquired and are illustrated in the figure to reflect the full data collection process; however, these data were not incorporated into the image analysis pipeline.

An overview of the proposed envisioned system is illustrated in Fig. 1.

A pilot study (ClinicalTrials.gov Identifier: NCT05044780, registered on 09/14/2021) was conducted to collect hyperspectral fingerprint images from adult participants (age $\ge$ 18 years) across two cohorts. Cohort 1 included individuals who tested positive for SARS-CoV-2 via standard-of-care molecular testing (PCR or antigen) within 7 days of enrollment; home test results were not accepted. Cohort 2 consisted of individuals with a confirmed negative SARS-CoV-2 test result within the same time frame, as determined by approved clinical methods. Exclusion criteria included participants who had received remdesivir and/or dexamethasone for more than 48 hours before hyperspectral imaging for COVID-19 treatment. Those who had received up to 48 hours of treatment remained eligible for the study. Optional laboratory evaluations included inflammatory cytokine panels and routine clinical labs (CBC, chemistry, hepatic, and mineral panels) drawn within 7 days of enrollment. HSI was performed using a touchless system on the right and left index fingers of each participant. Each finger was imaged three times to ensure complete surface coverage and reduce the risk of data artifacts. The imaging procedure took approximately 10 minutes per participant. *The negative samples were collected at the NIH site, while the positive samples were obtained at Inova*. Although instrument settings were adjusted to maintain similar parameters across both locations, differences in environmental conditions may have influenced the collected images. Using the same white reference tile in both rooms helps normalize illumination by providing a consistent basis for reflectance calibration. However, differences in ambient lighting (spectrum, intensity, angle), room geometry, reflections, and shadows can still cause subtle variations in the spectral data. While white tile calibration reduces some variability, it cannot eliminate environmental effects such as temperature, humidity, or differences in indirect lighting. Therefore, the same white reference improves comparability but does not ensure entirely consistent hyperspectral measurements across different rooms (Table 1).

**Table 1:** Demographic characteristics of study participants, including age, sex, race, and ethnicity distributions. Cohort 1 (n=3) includes COVID-19 positive subjects, and Cohort 2 (n=50) includes COVID-19 negative subjects, for a total of 53 individuals. (n indicates the number of subjects in each cohort).

	Cohort 1 (n=3)	Cohort 2 (n=50)	Total (n=53)
Age, years (median, range)	27.9 (18.4–44.8)	23.3 (21.9–71.0)	23.3 (18.4–71.0)
Female, n (%)	2 (66.7)	34 (68.0)	36 (67.9)
Race, n (%)			
White	3 (100)	29 (58.0)	32 (60.4)
Black/African American	–	2 (4.0)	2 (3.8)
Asian	–	11 (22.0)	11 (20.8)
American Indian	–	1 (2.0)	1 (1.9)
Multiracial	–	4 (8.0)	4 (7.5)
Unknown race	–	3 (6.0)	3 (5.7)
Ethnicity, n (%)			
Not Hispanic or Latino	3 (100)	43 (86.0)	46 (86.8)
Hispanic or Latino	–	5 (10.0)	5 (9.4)
Ethnicity not reported	–	1 (2.0)	1 (1.9)
Unknown	–	1 (2.0)	1 (1.9)

### HSI fingerprint analysis reveals two different spectral profiles for COVID-19 positive vs. negative patients

This dataset comprises hyperspectral fingerprint images from 53 subjects, 50 COVID-19 negative and 3 COVID-19 positive. For the negative class, we collected three samples per subject, resulting in a total of 150 samples. In contrast, the sampling for positive subjects was inconsistent during data collection. However, we were able to recover .hdr files for 6 usable positive samples.

The missing.hdr file was reconstructed by estimating the image dimensions (lines and rows) from the corresponding.bil file size, using the known number of spectral bands and data type to ensure consistency. To verify the integrity of the reconstruction, we performed a visual inspection of representative spectral signatures, confirming correct band ordering and alignment. This ensured that the recovered datacubes preserved smooth and physically consistent spectral profiles without introducing spectral shifts or artifacts.

Each hyperspectral cube captures spatially resolved spectral data across predefined wavelengths, reflecting physiological changes that may be linked to COVID-19. The dataset includes both symptomatic and asymptomatic individuals, supporting the identification of biochemical patterns distinguishing positive from negative cases.

**Fig. 2:**
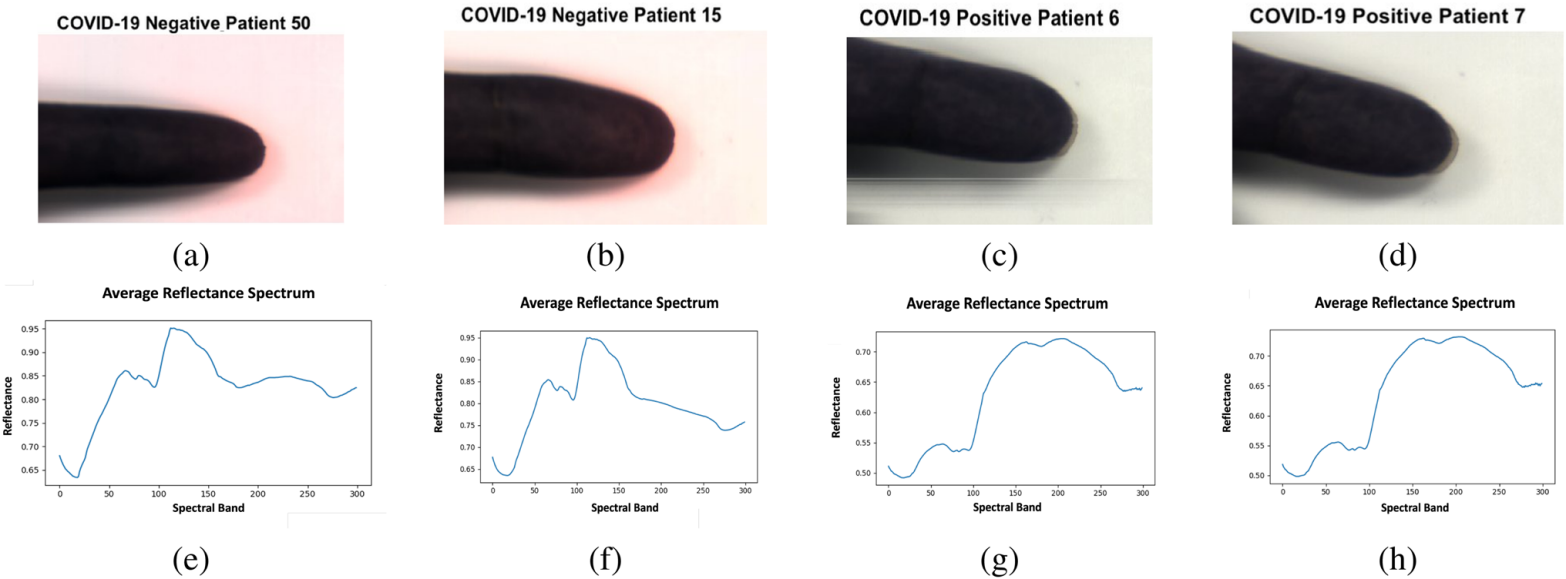
Fingertip spectral profiles: (**a**) and (**b**) correspond to two COVID-19 positive cases, with (**e**) and (**f**) showing their average reflectance across the 300 spectral bands acquired using the Resonon Pika-L hyperspectral imaging system. (**c**) and (**d**) correspond to two COVID-19 negative cases, with (**g**) and (**h**) presenting their average reflectance. Profiles are derived from HSI fingerprint data collected across four subjects.

Fig. 2 presents example hyperspectral fingerprint images from individuals classified as COVID-19 positive and negative. The average reflectance profiles exhibit consistent patterns among samples within the same class, suggesting potential spectral similarities related to infection status. To enable visual interpretation of hyperspectral data, we employed the function from the Spectral Python (SPy) library to generate pseudo-RGB images^9^. This function constructs a standard three-channel image by mapping selected spectral bands to the red, green, and blue channels, respectively. Given that hyperspectral images capture reflectance values across a wide range of wavelengths, often extending beyond the visible spectrum, direct visualization is not possible. By selecting bands within or near the visible range, pseudo-RGB representations offer an interpretable approximation of the scene and facilitate comparative analysis across samples. This approach supports the identification of patterns and anomalies that may not be apparent from numerical spectra alone.

We analyze hyperspectral signatures to distinguish between COVID-19-positive and negative classes. Reflectance curves across the full spectral range are visualized to identify characteristic differences between the two groups.

While the pushbroom camera captures full spatial-spectral information $(x, y, \lambda )$, our application focuses on the underlying biochemical reflectance properties of the fingertip rather than fine-grained spatial morphology. The goal of the study is to capture consistent spectral absorption and reflection patterns associated with physiological characteristics, which are preserved under spatial averaging. Although spatial texture information is not explicitly modeled, this loss is acceptable in our context, as the discriminative signal primarily resides in the spectral domain rather than in fine spatial structures.

**Fig. 3:**
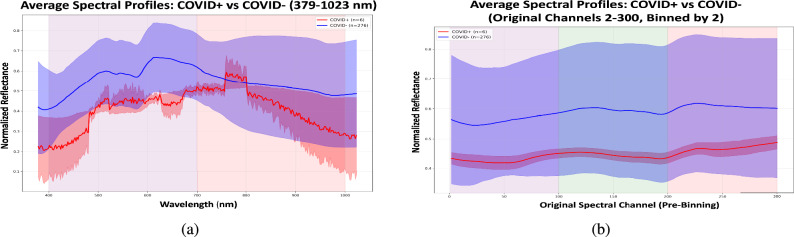
Average spectral signatures across all subjects: (**a**) full set of 300 spectral bands, and (**b**) a reduced set of 150 alternatively selected bands. The wavelength range is defined by the Resonon Pika-L sensor used for data collection.

Figure 3 (a) illustrates the average reflectance values across all 300 spectral bands for both COVID-positive and negative cases. The plot shows distinct spectral patterns between the two groups, with noticeable differences in specific wavelength ranges. This separation in reflectance profiles highlights the underlying physiological variations captured by the hyperspectral data. It supports the potential of these spectral features as reliable markers to distinguish the status of COVID-19. Distinct spectral patterns are observed between the two classes, particularly in specific wavelength ranges, indicating underlying physiological differences effectively captured by the hyperspectral imaging data. The consistent divergence in reflectance values across multiple bands supports the potential of spectral features as reliable indicators for binary classification in the context of COVID-19 detection. Figure 3 (b) illustrates further improvement in class separation achieved by selecting 150 alternatively chosen bands.

**Fig. 4:**
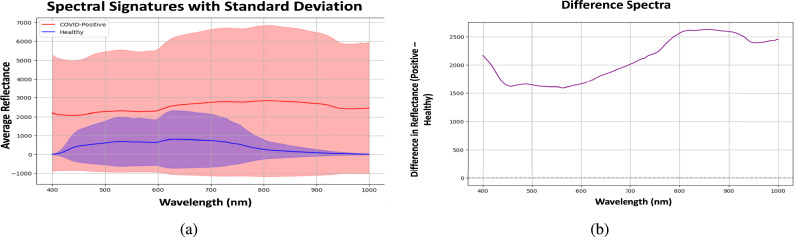
Average spectral signatures across all subjects: (**a**) average spectral signatures with variance, illustrating the average reflectance and variability across wavelengths, and (**b**) difference spectra, highlighting the wavelength-wise differences between groups. The wavelength range is defined by the Resonon Pika-L sensor used for data collection.

Figure 4(a) presents the average reflectance spectra for COVID-positive (red) and negative (blue) fingerprint samples. The shaded areas indicate one standard deviation, illustrating the variance within each group. Figure 4(b) shows the difference spectra, representing the wavelength-wise difference between the average reflectance of COVID-positive and negative samples. This highlights spectral regions with notable deviations that may correspond to physiological changes.

**Fig. 5:**
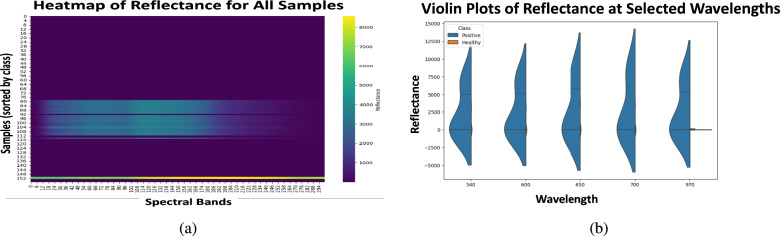
Reflectance distribution across all subjects: (**a**) heatmap of reflectance values for all samples across the full set of available spectral bands, and (**b**) violin plots of reflectance at selected wavelengths, illustrating the distribution and density of spectral responses.

Figure 5 illustrates (a) heatmaps of reflectance values across spectral bands for all samples, grouped by COVID-positive and negative classes to reveal patterns and variability, and Figure 5 (b) violin plots depicting the distribution of reflectance at selected wavelengths, highlighting differences between the two groups.

We also performed statistical significance tests to isolate the most informative spectral bands for the classification task. These significant bands form the basis for optimizing AI models, improving both the efficiency and accuracy of COVID-19 detection by focusing on the most discriminative wavelengths.

### Discriminative analysis identifying statistically significant spectral bands

**Fig. 6:**
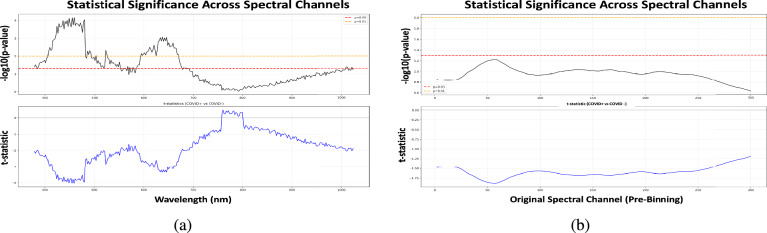
Statistical significance of spectral bands for COVID-19 classification: (**a**) using the full set of bands, and (**b**) after pre-binning, highlighting wavelengths that contribute most significantly to distinguishing COVID-19 positive and negative cases.

Fig. 6 (a) presents the results of statistical significance testing, which identified specific spectral regions with strong discriminative power. Bands between 680 nm and 1000 nm were significant at the $p = 0.05$ level, indicating moderate evidence against the null hypothesis. More importantly, bands from 380 to 400 nm, 500 to 680 nm, and at 1000 nm showed even stronger significance at the $p = 0.01$ level, implying only a 1% probability that these differences occurred by chance. These statistically significant bands, especially within the visible and near-infrared ranges, offer valuable guidance for model optimization by pinpointing the most informative wavelengths for COVID-19 classification. The statistical significance of the bands improves after pre-binning, as shown in Fig. 6 (b).

### Enhanced class separability

Principal Component Analysis (PCA) was applied to reduce dimensionality and enhance class separability, revealing distinct class-specific spectral patterns.

To prevent information leakage, PCA was fit on the training data within each validation fold, and the resulting transformation was applied to the corresponding test data.

**Fig. 7:**
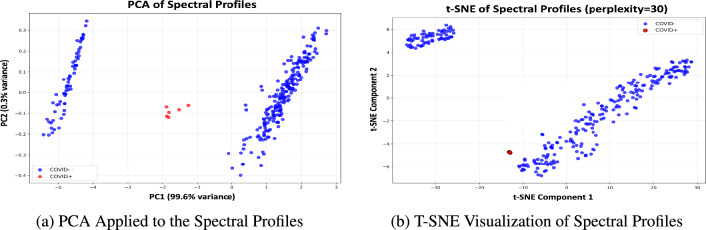
Dimensionality reduction of spectral data: (**a**) PCA projection, showing the main axes of variance in the data, and (**b**) t-SNE visualization, highlighting clustering patterns and nonlinear relationships among samples.

Fig. 7 (a) shows the resulting PCA projections, which demonstrate a clear differentiation between COVID-positive and negative samples in the reduced feature space. This separation indicates that the principal components effectively preserve class-specific spectral information, reinforcing the discriminative power of hyperspectral data even after dimensionality reduction. The PCA visualization thus confirms the distinct spectral characteristics of each class and supports the feasibility of using dimensionally reduced data for accurate and efficient classification. T-Distributed Stochastic Neighbor Embedding (t-SNE) was applied to visualize the high-dimensional spectral data in a two-dimensional space.

t-SNE parameters were set as follows: perplexity = 30, learning rate = 200, 1000 iterations, with a fixed random seed for reproducibility. Stability of the embeddings across multiple seeds was qualitatively verified. The t-SNE plots, generated with multiple random states, consistently demonstrated a separation between the positive and negative sample clusters. While the exact spatial arrangement varied slightly due to the non-deterministic nature of t-SNE, the distinct clustering of the two classes remained stable across all runs, reinforcing the potential for separability in lower dimensions.

The resulting projections, shown in Fig. 7 (b), reveal a distinct clustering of COVID-positive and negative samples, indicating strong class separability. Unlike PCA, t-SNE captures local and non-linear relationships in the data, further highlighting the discriminative structure of the spectral features. This visualization supports the potential of hyperspectral biometrics for accurate classification, even in reduced dimensions.

The chosen t-SNE parameters were selected to balance local structure preservation and stable visualization, given the highly imbalanced dataset (50 negative and 3 positive samples) and the small overall sample size. The perplexity was set to 30 to reflect a moderate neighborhood size, which is appropriate for a dataset of this scale, where values in the range of 5–50 are generally recommended^10^. A medium perplexity (20–50) balances local and slightly more global structure, making it a common choice in practice. This setting enables the method to capture meaningful local relationships among the majority class (negative samples) while maintaining overall structure, without overfitting to noise introduced by the very limited positive class. The learning rate of 200 was selected as a standard, robust value that supports convergence without causing instability. For datasets of this size, a moderate learning rate helps avoid overly tight clustering or fragmentation of embeddings, which can be particularly problematic when one class is underrepresented. The maximum number of iterations was set to 1000 to ensure sufficient optimization for convergence. Given the small dataset, this number of iterations allows the embedding to stabilize while still being computationally efficient. Finally, running t-SNE with multiple random seeds was necessary due to the stochastic nature of the algorithm. This helps assess the robustness and consistency of the observed clustering patterns, which is especially important in the presence of class imbalance and limited positive samples.

**Fig. 8:**
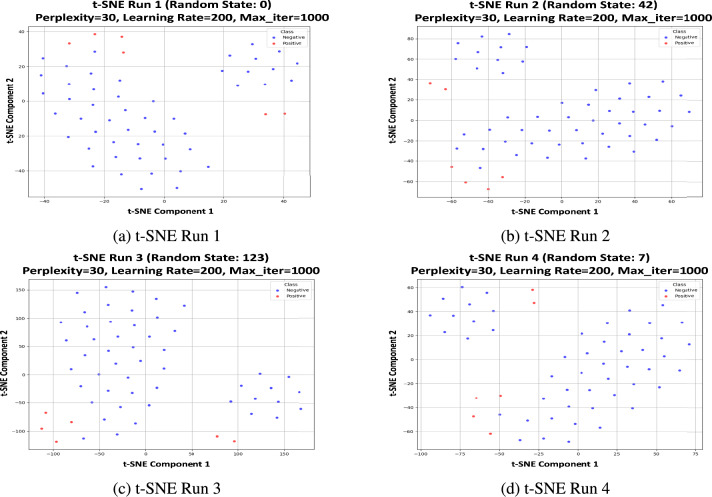
t-SNE visualizations of spectral profiles for four independent runs: each subplot shows the 2D embedding obtained with a different random seed, highlighting variability across runs. Parameters used: perplexity = 30, learning rate = 200, and maximum number of iterations = 1000.

The PCA (Fig. 7 (a)) and t-SNE plots (Figs. 7 (b) and 8 (a-d)) also reveal two distinct sub-clusters within the negative class. This structure may reflect inherent intra-class variability arising from differences in physiological characteristics, as well as minor variations in acquisition conditions and environmental or sensor-related factors. Such factors can introduce subtle differences in the spectral signatures, leading to separable groupings even within the same class. A detailed characterization of these sub-clusters is beyond the scope of this work.

### Classification of the COVID-19 fingerprint spectral profiles

Table 2 summarizes the dataset composition for our study, including the number of subjects per class, the number of acquisitions and hyperspectral samples per subject, and the final number of samples used for each experiment. A training-testing partition was applied, balanced to approximately 70% training and 30% testing, with subjects mutually exclusive between the two sets. Performance is reported at the sample level with subjects being mutually exclusive between training and testing partitions.

Because the test set contains 83 COVID-negative but only 2 COVID-positive samples, aggregate metrics such as overall accuracy must be interpreted with caution. In such an imbalanced and extremely small minority-class setting, a high accuracy value may primarily reflect correct classification of the majority class rather than stable detection of COVID-positive cases. For this reason, we report class-specific precision, recall, and F1-score, together with averaged summaries, to provide a more transparent view of minority class behavior.

**Table 2:** Dataset accounting and sample flow across experiments.

Class	Subjects	Samples per Subject	Training	Testing
COVID-19 Negative	50	3 RxIx (all subjects) + 3 LxIx (all subjects)	217 (72.3%)	83 (27.7%)
COVID-19 Positive	3	2 LxIx (2 subjects) + 1 RxIx (1 subject) + 1 LxIx (1 subject)	4 (66.7%)	2 (33.3%)
Total	53	300 negative + 6 positive = 306	221 (72.2%)	85 (27.8%)

We report results at the subject level (see Tables 3 and 4). In this setting, negative samples are partitioned by sample across six different runs, while positive samples remain unchanged across runs and sample types (left or right index, and impression number) due to the limited number of positive images available in the dataset.

**Table 3:** Subject-level SVM classification performance over five random runs.

Setting	Avg. Accuracy ± Std.	Macro Prec.	Macro Recall	Macro F1	W. Prec.	W. F1
LxIx1	0.884 ± 0.070	0.542	0.600	0.569	0.786	0.831
LxIx2	0.922 ± 0.096	0.761	0.800	0.778	0.860	0.888
LxIx3	0.952 ± 0.057	0.676	0.700	0.688	0.907	0.928
RxIx1	0.964 ± 0.053	0.782	0.600	0.791	0.932	0.947
RxIx2	0.931 ± 0.070	0.665	0.700	0.682	0.870	0.898
RxIx3	0.931 ± 0.084	0.672	0.700	0.681	0.872	0.899

**Table 4:** Subject-level logistic regression classification performance over five random runs.

Setting	Avg. Accuracy ± Std.	Macro Prec.	Macro Recall	Macro F1	W. Prec.	W. F1
LxIx1	0.976 ± 0.026	0.887	0.867	0.873	0.965	0.968
LxIx2	0.956 ± 0.090	0.878	0.900	0.888	0.921	0.936
LxIx3	0.879 ± 0.050	0.735	0.647	0.659	0.852	0.847
RxIx1	0.910 ± 0.094	0.655	0.700	0.676	0.836	0.869
RxIx2	0.923 ± 0.074	0.859	0.807	0.815	0.902	0.902
RxIx3	0.898 ± 0.084	0.648	0.667	0.652	0.825	0.856

We also report, as a secondary evaluation, results obtained using all samples while keeping subjects mutually exclusive between the training and testing sets. Table 5 presents detailed SVM classification performance metrics obtained over five independent runs. For comparison, Table 6 presents detailed Logistic Regression classification performance metrics obtained over five independent runs.

**Table 5:** SVM classification performance over five random runs using sample-level evaluation with subject-exclusive training and testing.

Run	Accuracy	Macro Precision	Macro Recall	Macro F1	Weighted Precision	Weighted F1
1	1.000	1.000	1.000	1.000	1.000	1.000
2	1.000	1.000	1.000	1.000	1.000	1.000
3	0.938	0.469	0.500	0.484	0.879	0.907
4	1.000	1.000	1.000	1.000	1.000	1.000
5	0.913	0.457	0.500	0.477	0.834	0.872
**Avg**	**0.970 ± 0.042**	**0.785**	**0.800**	**0.792**	**0.942**	**0.956**

**Table 6:** Logistic Regression classification performance over five random runs using sample-level evaluation with subject-exclusive training and testing.

Run	Accuracy	Macro Precision	Macro Recall	Macro F1	Weighted Precision	Weighted F1
1	1.000	1.000	1.000	1.000	1.000	1.000
2	1.000	1.000	1.000	1.000	1.000	1.000
3	0.978	0.489	0.500	0.495	0.957	0.968
4	0.979	0.989	0.833	0.895	0.980	0.977
5	0.957	0.977	0.750	0.822	0.958	0.950
**Avg**	**0.983 ± 0.018**	**0.891**	**0.817**	**0.842**	**0.979**	**0.971**

While these results are promising, they are based on a limited pilot dataset with testing specifically consisting of 83 COVID-negative and only 2 COVID-positive samples. This small sample size limits the generalizability of the findings but demonstrates the potential of hyperspectral features for effective discrimination in controlled settings. Further validation with larger and more balanced datasets is needed to confirm the robustness of the approach.

We additionally report class-specific performance metrics for both COVID-19 negative and positive classes (Table 7). These results provide further insights beyond aggregate metrics and highlight the impact of class imbalance. In particular, Runs 1 and 4 demonstrate that the model fails to correctly identify positive cases (F1-score = 0.000), despite achieving high overall accuracy. This discrepancy emphasizes that accuracy and weighted metrics alone can be misleading in imbalanced settings. The reported class-specific results therefore motivate the use of macro-averaged metrics reported in all the tables above and reinforce the importance of evaluating per-class performance for a more reliable and transparent assessment.

**Table 7:** Class-specific performance for the RO3 sample (Logistic Regression) across five runs. We report precision (P), recall (R), and F1-score (F1) for COVID-19 negative (0) and positive (1) classes.

Run	Class 0 (COVID-19 Negative)			Class 1 (COVID-19 Positive)		
	P	R	F1	P	R	F1
Run 1	0.938	1.000	0.968	0.000	0.000	0.000
Run 2	0.977	1.000	0.988	1.000	0.667	0.800
Run 3	0.977	1.000	0.988	1.000	0.667	0.800
Run 4	0.913	1.000	0.955	0.000	0.000	0.000
Run 5	1.000	1.000	1.000	1.000	1.000	1.000

## Discussion

The proposed HSI-based analysis system holds promise for integration into routine inpatient and outpatient workflows. Unlike traditional methods, this system may ultimately be able to provide real-time Clinical Decision Support (CDS) when the relevant anatomical region is properly captured, enabling proactive risk assessment through early detection of surface changes due to inflammation, of infectious or other origin, perhaps by detecting biomarkers of inflammation in sweat. Our data indicate that spectral signatures may be capable of distinguishing between COVID-19-negative and COVID-19-positive individuals, potentially distinguishing early physiologic changes. The average spectral profiles of the two classes are distinctly separable. In line with our findings, other studies showed that HSI captures pixel-wise spectral information that enables the mapping of oxyhemoglobin (HbO_2_) and deoxyhemoglobin (Hb) concentrations in tissue. This capability makes HSI well-suited for non-invasively estimating blood oxygen saturation (SpO_2_)^11^, a critical metric for assessing COVID-19 severity^12^. For instance, non-contact diffuse-reflectance HSI (400–980 nm) of the volar forearm in healthy volunteers produced skin oxygen saturation (StO_2_) values within the uncertainty range of SpO_2_ measured by clinical fingerprint pulse oximeters^11,13^. In critically ill COVID-19 patients, bedside HSI monitoring revealed persistently reduced superficial tissue oxygenation (StO_2_) and Near-infrared Perfusion Index (NPI), with shifts in hemoglobin absorption spectra that correlated with organ dysfunction scores^12^. Collectively, these studies underscore the potential of HSI to detect spectral biomarkers of an inflammatory physiology, and be developed as a non-invasive and real-time test. For effective interpretation, analysis after normalization should focus on key spectral regions relevant to diagnostics: 600–700 nm (oxyhemoglobin), 540–600 nm (deoxyhemoglobin), and approximately 970 nm (inflammation indicated by water content).

Inflammation may influence spectral signatures captured by hyperspectral imaging through changes in skin composition, blood perfusion, and sweat metabolite content. COVID-19, in particular, is known to disrupt metabolic pathways, immune function, and vascular integrity–factors that can alter the biochemical composition of sweat and thus affect the spectral profiles acquired from the fingerprint. Given the sensitivity of hyperspectral sensors to tissue oxygenation, water content, and molecular variation, a biologically driven difference between COVID-19-positive and COVID-19-negative samples is plausible. However, the potential influence of environmental variables, such as ambient lighting, temperature, humidity, and sensor positioning, cannot be excluded. Hyperspectral imaging is especially susceptible to changes in scene illumination, and data collected at different physical locations may reflect not only biological differences but also environmental or instrumental variability. In this study, positive and negative samples were collected at separate sites (Inova and NIH, respectively), which may have introduced systematic bias. Since the sensor has its lighting and camera operated with cutoff filtering, the effect of environmental light is minimal. Future studies should standardize acquisition protocols and collect all class samples in the same physical environment under controlled conditions.

This early pilot study demonstrates the potential of HSI to non-invasively detect COVID-19 infection by capturing subtle biochemical and physiological changes in the skin and creates the foundation for a larger prospective multi-center clinical trial. Integrating HSI with AI can enable a novel paradigm in medical imaging toward earlier and more precise identification of infectious diseases and other inflammatory states. While further validation is needed, the HSI-based analysis system may be developed further for integration into routine inpatient and outpatient workflows and enable real-time clinical decision support and proactive risk assessment by detecting early signs of inflammation or infection. As a pilot effort, this work lays the foundation for future bioengineering research and the development of next-generation diagnostic tools.

## Methods

Imaging was performed using a Resonon Pika L pushbroom (line-scan) camera, which captures one line of pixels at a time to build a 3D datacube $(x, y, \lambda )$. Compared to point-scan systems, pushbroom sensors provide faster data acquisition without sacrificing spectral resolution^14–16^. The HSI system acquires both spatial and spectral information across a broad wavelength range^17,18^. A lens system collects reflected light, focuses it through a narrow slit, and disperses it by a diffraction grating. An array of wavelength-sensitive photo-detectors then converts the dispersed spectral data into electrical signals. The primary components of the system include: (1) focusing lenses, (2) a beam-defining slit, (3) a diffraction grating, and (4) photo-detectors for spectral measurement^19^. Each participant had their right and left index fingers imaged by the camera using a touchless system. Each finger was imaged three times to ensure proper acquisition of the entire finger surface and to reduce the risk of unclear data. The imaging session took approximately 10 minutes. The *reference tile* was used following standard procedures for hyperspectral imaging. It was placed on the imaging stage beneath the lighting system to provide a stable and uniform reflectance reference. Participants positioned their hands at predefined, fixed locations on the reference tile during each acquisition, ensuring consistent illumination and minimizing variability across captures. The protocol was approved by the NIH Institutional Review Board (IRB) and the INOVA Human Subject Research Protection Program (HRPP). Informed consent was obtained from all participants.

### Pre-processing

We analyze *skin reflectance* in the hyperspectral domain, leveraging the technology’s ability to capture detailed biochemical and physiological information. Given the sensitivity of hyperspectral imaging to subtle changes in tissue composition and oxygenation, we expect it to detect spectral signatures associated with COVID-19 infection. This capability opens the possibility of non-invasive monitoring and early detection of disease through characteristic alterations in skin reflectance patterns^20–23^. All hyperspectral data were first converted from raw radiance to reflectance using a standard calibration procedure:

1 $$\begin{aligned} R = \frac{I_\text {sample} - I_\text {dark}}{I_\text {white} - I_\text {dark}}, \end{aligned}$$

where $I_\text {sample}$ is the raw measurement, $I_\text {dark}$ is the dark reference, and $I_\text {white}$ is the measurement of a certified reference tile^24^. The reference tile was measured prior to each session to ensure stability. Spectral standardization was applied per wavelength channel using statistics computed only on the training data to prevent information leakage. Noisy or missing bands, including saturated or low-SNR regions, were removed prior to analysis, and any remaining gaps were linearly interpolated. To obtain a robust and concise spectral signature for each subject, we extracted the *average reflectance* values by averaging across the spatial dimensions of each hyperspectral image. This approach captures the general characteristics of the skin reflectance while minimizing the influence of local variations and noise. The resulting averaged reflectance profile served as the input for subsequent analysis and classification.

### Dimensionality reduction

Dimensionality reduction was performed using Principal Component Analysis (PCA) to reduce the spectral feature space while retaining 95% of the variance. PCA reduces dimensionality by projecting data onto components that capture the most variance, facilitating visualization and highlighting class-specific spectral patterns^25^. In addition, t-distributed Stochastic Neighbor Embedding (t-SNE) was used to produce a non-linear low-dimensional embedding, preserving local structure and enhancing differentiation of classes.

### Classification

To evaluate the ability of hyperspectral skin reflectance profiles to distinguish COVID-19 positive from negative cases, we trained supervised machine learning classifiers in subject-level spectral data. Our goal was to capture subtle, potentially non-linear differences in the spectral signatures between classes while ensuring robust generalization across individuals. We considered both traditional linear models and non-linear classifiers to benchmark performance and provide complementary perspectives on the separability of the data.

A *Support Vector Machine (SVM)* classifier with a nonlinear Radial Basis Function (RBF) kernel was trained to distinguish between COVID-19-positive and negative cases, capturing complex relationships in the spectral feature space^25^:

2 $$\begin{aligned} K(\textbf{x}_i, \textbf{x}_j) = \exp \left( -\gamma \Vert \textbf{x}_i - \textbf{x}_j\Vert ^2\right) \end{aligned}$$

The dataset was split into training and testing subsets using a 70/30 ratio,

as indicated in Table 2.

A *subject-exclusive* partitioning strategy ensured that no subject appeared in both training and testing sets, enabling robust evaluation of model generalization across individuals.

A *Logistic Regression* model was also trained on the spectral profiles, estimating the probability of COVID-19 positivity via the sigmoid function:

3 $$\begin{aligned} P(y=1|\textbf{x}) = \frac{1}{1 + e^{-(\textbf{w}^\top \textbf{x} + b)}} \end{aligned}$$

where $\textbf{x}$ is the input feature vector, $\textbf{w}$ the weight vector, and $b$ the bias term^25^.

All experimental procedures were performed according to relevant institutional, national, and international guidelines and regulations.

## Limitations

The limitation of this pilot study is the small number of positive samples in the dataset, with only three individuals in the COVID-19 positive group. Although there is a clear separability between the two classes, our observations may not be fully representative of the positive class.

Since the spectral profiles appear to be non-linearly separable in both the PCA and t-SNE projections, we employ an SVM with a non-linear RBF kernel to effectively handle this non-linearity.

This work highlights the inherent difficulties in binary classification, especially under conditions of class imbalance and small sample sizes, which can impact model performance and generalizability. Moreover, positive and negative samples were collected at separate sites due to practical constraints, including limited availability of positive cases during peak pandemic waves, which may introduce site-related variability. To mitigate this, both sites followed identical acquisition protocols with fixed illumination and controlled indoor settings, for the learned patterns primarily reflect physiological differences rather than environmental or procedural artifacts. Future studies should standardize acquisition protocols and collect all class samples in the same physical environment under controlled conditions. This will help to disentangle the contributions of biological signals from external factors, thereby improving the reliability of hyperspectral biomarkers for disease detection.

While the data collection was conducted under controlled conditions and direct illumination was normalized, minor environmental scattering differences between the two clinical settings remain a confounding factor.

On a larger dataset with more positive samples, key steps to accurately analyze skin reflectance data include: first, normalizing the sample reflectance by dividing it by the reflectance of a shadow-free white tile to eliminate lighting artifacts, especially the pronounced red signal from the light source. Accurate spectral analysis requires careful calibration using white and dark references to correct for variations in illumination and sensor noise. Although HSI cameras capture data across hundreds of spectral bands, dimensionality reduction techniques are commonly employed to simplify the dataset while preserving critical information^26^. By enabling rapid, contactless assessment of molecular skin signatures, HSI presents a scalable solution for infectious disease screening in both clinical and public health settings. Nevertheless, the high dimensionality of HSI data introduces challenges such as spectral redundancy and the need to maintain spectral structure integrity. Addressing these challenges demands advanced, tailored dimensionality reduction and feature extraction methods to leverage the capabilities of HSI technology fully^20,27^. The fingertip is a practical site for hyperspectral imaging due to its ease of access, low motion, and established biometric use, offering a user-friendly alternative to facial regions, where HSI may raise usability or privacy concerns.

## Conclusions

This work introduced a novel pilot framework for COVID-19 detection based on one-dimensional reflectance-derived spectral signature profiles, focusing on the feasibility of leveraging such signals for classification. The proposed pipeline demonstrates promising performance, with high overall accuracy across multiple runs; however, class-specific analysis reveals variability in detecting the minority (positive) class, underscoring the challenges posed by limited and imbalanced data. These findings highlight the importance of incorporating per-class evaluation to ensure transparent and reliable assessment. While the current study is constrained by the small sample size and the use of 1D spectral data, it establishes a foundation for future research. In particular, expanding the dataset, and improving robustness to class imbalance will be critical next steps to validate and extend the proposed methodology. Our classification findings remain difficult to interpret. In this pilot study, Support Vector Machine (SVM) and Logistic Regression analyses provided preliminary evidence that hyperspectral fingertip signatures may contain discriminatory information related to COVID-19 status. However, because the positive cohort was extremely limited, these results should be interpreted strictly as feasibility findings rather than reliable estimates of generalizable diagnostic performance.

## Data Availability

The datasets used and/or analyzed during this study are available from the corresponding author upon reasonable request. In particular, the raw hyperspectral cubes with associated header files will be released and made available on Zenodo prior to the manuscript’s publication.
